# Impact of plant monoterpenes on insect pest management and insect-associated microbes

**DOI:** 10.1016/j.heliyon.2024.e39120

**Published:** 2024-10-09

**Authors:** Muhammad Qasim, Waqar Islam, Muhammad Rizwan, Dilbar Hussain, Ali Noman, Khalid Ali Khan, Hamed A. Ghramh, Xiaoqiang Han

**Affiliations:** a Key Laboratory of Oasis Agricultural Pest Management and Plant Protection Utilization, College of Agriculture, Shihezi University, Shihezi, 832002, Xinjiang, China; b Xinjiang Key Laboratory of Desert Plant Roots Ecology and Vegetation Restoration, Xinjiang Institute of Ecology and Geography, Chinese Academy of Sciences, China; c Department of Entomology, University of Agriculture, Faisalabad, Sub-campus Depalpur, Okara, 56300, Pakistan; d Department of Entomology, Ayub Agricultural Research Institute, Faisalabad, 38850, Pakistan; e Department of Botany, Government College University Faisalabad, Faisalabad, 38040, Pakistan; f Applied College, Center of Bee Research and its Products, Unit of Bee Research and Honey Production, and Research Center for Advanced Materials Science (RCAMS), King Khalid University, P.O. Box 9004, Abha, 61413, Saudi Arabia

**Keywords:** Antimicrobial, Environmental safety, Insect control, Insect repellant, Monoterpenes, Organic farming, Public health

## Abstract

The fight against insect pests primarily relies on the utilization of synthetic insecticides. However, improper application of these chemicals can lead to detrimental effects on both the environment and human health, as well as foster the development of insect resistance. Consequently, novel strategies must be implemented to address the challenges stemming from the prolonged use of synthetic insecticides in agricultural and public health environments. Certain strategies involve the combination of crop protectants, which not only enhance insecticidal effectiveness but also reduce application rates. Plant-based natural products emerge as promising alternatives for insect management. Monoterpenes, which are abundant plant compounds produced through the activation of various enzymes, have attracted significant attention for their effectiveness in insect control. Notably, they are prolific in fragrance-producing plants. This review explores the plant defense, insecticidal, and antimicrobial characteristics of monoterpenes against insect pests, shedding light on their potential modes of action and possibilities for commercialization. Emphasizing their role as targeted and environmentally safer, the review highlights the practical viability of monoterpenes within integrated pest management programs.

## Highlights

• Improper application of synthetic insecticides causes environment and human health issues and develops insect resistance.
• Botanical insecticides are needed to safe environment and public health.
• Monoterpenes are promising alternatives for insect management.
• Monoterpenes are lethal to insect pests, and are insect repellant and antifeedants.
• Monoterpenes are effective certain microbes.

## Graphical abstract

**Figure:**
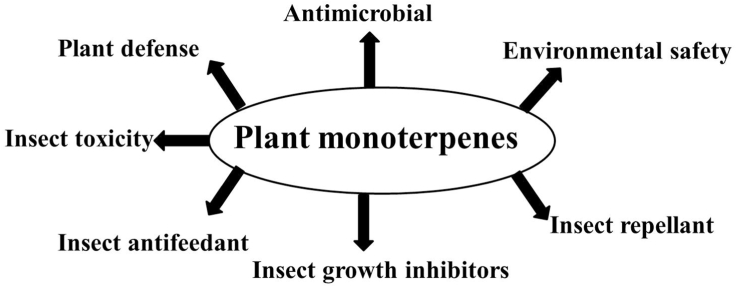

## 1 Introduction

Plants represent a rich source of secondary metabolites, including antioxidants such as ascorbic acid, flavanones, and simple phenolics, as well as folate and pectin, which are vital for human nutrition. Limonoids, present mostly in Meliaceae and Rutaceae families-including citrus plant parts, are another category of secondary metabolites whose significance in human nutrition remains unclear [1]. These triterpenoid compounds manifest in citrus fruits as either limonoid aglycones or limonoid glycosides. Limonoid glucosides are abundant in the fruits and juices of plants [2]. Considerable quantities of limonoid glucosides have been documented in byproducts resulting from citrus processing, with an estimated global availability of 15,000 tons [3]. Limonoid glycosides, such as limonin glucoside, are water-soluble, tasteless, non-mutagenic [4], and have demonstrated no significant toxic effects when included in animal diets [5]. On the other hand, limonoid aglycones, like limonin, are present in citrus juices at low concentrations. Some of these water-insoluble aglycones are accountable for the emergence of delayed bitterness in citrus juices, even at concentrations as minimal as 6 ppm [6].

Flavonoids belong to a group of naturally-occurring polyphenolic compounds distinguished by a shared benzo-γ-pyrone structure. They play a crucial role in crops, fruits, and vegetables [7]. Over 8000 compounds with a flavonoid structure have been identified. The considerable quantity results from the diverse combinations of multiple hydroxyl, methoxyl, and O-glycoside group substituents on the fundamental benzo-γ-pyrone structure (C_6_-C_3_-C_6_) [8].

Fruits and their derivatives from plants offer significant health benefits due to their nutritional and antioxidant properties. Their consumption is linked to reduced cardiovascular disease incidence and a lower risk of specific cancers. In the processing of plant fruits for juice extraction, peels emerge as the primary by-product, rich in phytochemicals. These compounds (nerol, estragole, α-terpinene, ocimene, farnesene, pulegone, and 3,7-dimethyl-1-octanol) are renowned for their antioxidant activity and ability to scavenge free radicals, contributing to the health-promoting qualities associated with plant peels [[9], [10], [11], [12]].

Numerous studies have extensively explored phenolic compounds and antioxidant activities within citrus peel, revealing a spectrum of health benefits including anti-allergenic, anti-atherogenic, anti-inflammatory, antimicrobial, antioxidant, antithrombotic, cardioprotective, and vasodilatory effects [[13], [14], [15]]. Moreover, the chemical composition plays a pivotal role in elucidating plants' varied responses to external stresses, whether biotic or abiotic. Consequently, researchers have delved into the variability of bioactive compounds, their interplay with genetic and climatic factors, and their implications for plant taxonomy, as evidenced by previous works [[16], [17], [18], [19], [20]]. Predominant constituents comprise flavonoids, notably polymethoxylated flavonoids, terpenoids like limonene and linalool, alongside other volatile oils [21].

## 2 Terpenes

Terpenes, the largest class of secondary metabolites, share a common biosynthetic origin from either acetyl-coA or glycolytic intermediates [[22], [23], [24]]. Most terpenes structures produced by plants as secondary metabolites are presumed to function in defense, acting as toxins and feeding deterrents against numerous plant-feeding insects and mammals [25,26]. Terpenes are categorized based on the number of isoprene units in the molecule, with a prefix indicating the requisite number of terpene units for assembly [27]. The primary groups of terpenes include Hemiterpenes, Sesquiterpenes, Diterpenes, Sesterterpenes, Triterpenes, Sesquarterpenes, Tetraterpenes, Polyterpenes, Norisoprenoids, and Monoterpenes.

### 2.1 Monoterpenes

Monoterpenes represent a category of terpenes composed of two isoprene units, denoting a molecular formula of C_10_H_16_. These organic compounds constitute a significant portion of the essential oils present in numerous plants, enriching them with distinctive aromas and flavors [[28], [29], [30], [31]]. Their multifaceted roles in plant ecology encompass defense mechanisms against herbivores and pathogens, attraction of pollinators, and allelopathic interactions [32,33]. Monoterpenes are synthesized from isoprene units (C_5_H_8_) via either the mevalonate pathway or the methylerythritol phosphate (MEP) pathway [34]. This biosynthetic process commences with the generation of the universal 5-carbon precursor, isopentenyl pyrophosphate (IPP), and its isomer, dimethylallyl pyrophosphate (DMAPP) [35]. These precursor compounds amalgamate to yield geranyl pyrophosphate (GPP), the 10-carbon precursor responsible for all monoterpenes [36]. The structure of monoterpenes assumes various forms, including acyclic (such as myrcene and geraniol), monocyclic (like limonene and menthol), or bicyclic (including pinene and camphor). Furthermore, monoterpenes are categorized into distinct groups, encompassing camphane, carane, cyclocitral, fenchane, iridoid, linear, menthane, ochtodane, pinane, seco-iridoid, and miscellaneous monoterpenes [37,38].

Monoterpenes are widely used in various industries due to their aromatic properties and biological activities. Their antimicrobial, anti-inflammatory, and analgesic effects make many monoterpenes essential in the field of medicine [39,40]. Utilization of menthol is employed in cough drops and topical analgesics [41,42]. Moreover, the pleasing fragrances of monoterpenes deem them indispensable components in perfumes, lotions, and various personal care formulations [43,44]. Additionally, monoterpenes serve as flavoring agents within the food and beverage sector. Limonene, for instance, is a prevalent choice for infusing citrus-flavored products with its distinct essence [45]. Furthermore, certain monoterpenes serve as natural pesticides and herbicides due to their efficacy in repelling insects and stunting plant growth.

Over 80,000 terpenes have been discovered within living organisms, with monoterpenes representing a significant class found abundantly in plants and essential oils. They exhibit a wide range of chemical structures and biological activities [46]. Monoterpenes are classified into regular, irregular, rearranged, and degraded types. Some adhere to Ruzicka's Isoprene Biogenetic Rule, while others follow different biogenetic pathways, resulting in artemisyl, chrysanthemyl, lavandulyl, and santolinyl skeletons [47]. The emission rates of monoterpenes from various fruits, plants, and vegetables have been measured. Common monoterpenes, include *α*-pinene, *β*-pinene, myrcene, *α*-terpinene, R-limonene, γ-terpinene, and p-cymene, with variations observed in emission profiles among different samples [48]. Furthermore, a phytochemical study identified five known monoterpenes in the leaves of *Rhododendron anthopogon*: ranhuadujuanine A, cannabiorcicyclolic acid, ranhuadujuanine B, ranhuadujuanine C, and ranhuadujuanine D, and some ^13^C-signals were revised based on 2D-NMR spectra [49,50].

Monoterpenes (*α*-pinene and limonene) play a crucial role in ecosystems, contributing significantly to the formation of secondary organic aerosols (SOAs) in the atmosphere [51]. These aerosols, influenced by the emission of monoterpenes from plants, have profound effects on cloud formation, climate regulation, and air quality.

Upon release into the atmosphere, monoterpenes undergo oxidation processes initiated by hydroxyl radicals (OH), ozone (O_3_), and nitrate radicals (NO_3_) [[52], [53], [54]]. This oxidation leads to the creation of SOAs, which in turn play a pivotal role in regulating Earth's radiative balance by scattering sunlight and acting as cloud condensation nuclei (CCN) [55,56]. Consequently, this process could potentially cool the climate by increasing cloud reflectivity. Such interactions form part of the complex biosphere-atmosphere feedback mechanisms crucial for climate regulation.

Moreover, monoterpenes influence various ecological dynamics. They affect plant-pollinator interactions and herbivore dynamics, thus contributing to biodiversity maintenance. Additionally, through allelopathy, monoterpenes influence plant community composition and ecosystem stability [57]. Understanding these ecological roles could guide sustainable agricultural practices, such as the use of monoterpene-based biopesticides to reduce reliance on synthetic chemicals [58,59]. Floral monoterpenes serve as attractants for pollinators, aiding in plant reproduction by signaling the presence of nectar and pollen. Linalool, a prevalent floral monoterpene, is particularly attractive to bees and silk moths [[60], [61], [62]].

Monoterpenes, part of the complex blend of volatile organic compounds (VOCs) emitted by plants under biotic stress, act as signals to neighboring plants, priming them for potential herbivore attacks by inducing the production of defensive compounds (*α*-terpineol, *β*-myrcene, bornyl acetate, carvacrol, linalool, and terpine-4-ol) [[63], [64], [65], [66]]. Furthermore, through various pathways like leaf litter, root exudation, or volatilization, monoterpenes inhibit the germination and growth of competing plant species. For example, compounds like *α*-pinene and camphor exhibit allelopathic properties, suppressing seedling growth and microbial activity in the soil [67,68].

Numerous studies suggest that monoterpenes primarily exert their insecticidal effects by targeting the nervous system of insects [69]. Like geraniol, citral, and eugenol have been shown to reduce spontaneous and irritating impulses from the ventral nerve cord cells [70]. Thymol disrupts the electrical activity of the dorsal longitudinal flight muscles, lowering flight muscle impulses and wing-beat frequency [71,72]. Linalool has neuroexcitatory properties, while eugenol exhibits neuroinhibitory effects [73]. Ion channels such as γ-aminobutyric acid type A receptors (GABA_A_Rs), nicotinic acetylcholine receptors (nAChRs), tyramine (TA) receptors, octopamine (OA) receptors, transient receptor potential (TRP) channels, as well as enzymes like acetylcholinesterase (AChE) and Na^+^/K^+^-ATPase, are potential targets for monoterpenes within the insect nervous system [74,75]. Ligand-binding assays reveal that carvacrol, thymol, and 1,8-cineole enhance the binding of t-butylbicycloorthobenzoate (TBOB) to GABA_A_Rs, while camphor inhibits this binding. Whole-cell patch-clamp recordings demonstrate that linalool suppresses GABA_A_R currents. Thymol acts as an orthosteric modulator of GABA_A_Rs, enhancing GABA-induced currents [76,77]. Carvacrol non-competitively inhibits the binding of nicotine to nAChRs, and linalool may similarly inhibit nAChR currents. Both carvacrol and thymol affect TA receptors, inhibiting the binding of tyramine. Eugenol and α-terpineol suppress the binding of octopamine to OA receptors [78,79]. Thus, several monoterpenes, including linalool, myrcene, citral, limonene, α-terpineol, terpinen-4-ol, γ-terpinene, carvacrol, thymol, p-cymene, α-pinene, and 1,8-cineole, inhibit AChE and Na+/K + -ATPase activities, suggesting that these enzymes may be primary targets of monoterpene action, and play as substantial biopesticides against different insect pests (Table 1).

**Table 1:** List of monoterpenes for pesticidal activities.

Monoterpene	Target insects	References
Monoterpenes as fumigants		
1,8-cineole	*Callosobrunchus maculatus, Culex pipiens, Culex quinquefasciatus, Plodia interpunctella, Reticulitermes dabieshanensis, Sitophilus granarius, Sitophilus oryzae, Tribolium confusum*	[[80], [81], [82], [83], [84], [85], [86]]
Borneol	*Callosobruchus chinensis*	[87]
Camphor	*Aphis nerii, Culex pipiens, Callosobruchus chinensis, Thrips palmi, Tuta absoluta*	[87,[88], [89], [90], [91]]
Carvacrol	*Anaphothrips obscurus, Bemisia tabaci, Musca domestica, Plutella xylostella, Sitophilus granarius, Tribolium castaneum, Trogoderma granarium*	[81,[92], [93], [94], [95], [96], [97]]
Citral	*Bemisia tabaci, Callosobrunchus maculatus, Culex quinquefasciatus, Plutella xylostella, Reticulitermes flaviceps, Sitophilus oryzae, Sitophilus zeamais*	[93,[98], [99], [100], [101], [102]]
Citronellal	*Anopheles gambiae, Bemisia tabaci, Culex pipiens, Drosophila suzukii, Plodia interpunctella, Plutella xylostella, Reticulitermes dabieshanensis, Reticulitermes flaviceps, Sitophilus oryzae, Tribolium castaneum*	[82,86,93,98,99,[103], [104], [105], [106]]
Carvone	*Culex pipiens, Reticulitermes dabieshanensis, Tenebrio molitor, Tribolium castaneum*	[84,[107], [108], [109]]
Cinnamaldehyde	*Culex pipiens*	[105]
Cuminaldehyde	*Aphis nerii, Plutella xylostella*	[89,93]
Estragole	*Callosobrunchus maculatus, Drosophila melanogaster*	[110,111]
Eucalyptol	*Callosobruchus chinensis*	[87]
Eugenol	*Bemisia tabaci, Bradysia procera, Cimex lectularius, Halyomorpha halys, Musca domestica, Plutella xylostella, Reticulitermes dabieshanensis, Sitophilus oryzae, Tuta absoluta*	[70,86,88,93,98,101,[112], [113], [114]]
Geranial	*Drosophila suzukii*	[104]
Geraniol	*Callosobrunchus maculatus, Culex quinquefasciatus, Reticulitermes flaviceps, Sitophilus oryzae, Sitophilus zeamais, Tribolium castaneum*	[99,100,102,103]
Limonene	*Aedes albopictus, Bemisia tabaci, Callosobrunchus maculatus, Drosophila melanogaster, Halyomorpha halys, Plodia interpunctella, Reticulitermes dabieshanensis, Sitophilus zeamais, Sitophilus oryzae, Tribolium confusum*	[65,82,83,108,112,[115], [116], [117], [118], [119]]
Linalool	*Callosobrunchus maculatus, Culex pipiens, Culex quinquefasciatus, Drosophila melanogaster, Plodia interpunctella, Sitophilus granarius, Sitophilus zeamais, Thrips palmi*	[120,81,82,90,102,105,111,121]
Menthol	*Aphis nerii, Halyomorpha halys, Reticulitermes dabieshanensis, Sitophilus oryzae*	[86,89,101,112]
Menthone	*Sitophilus granarius, Sitophilus oryzae, Thrips palmi*	[81,90,122]
Myrcene	*Callosobrunchus maculatus, Drosophila melanogaster, Lasioderma serricorne, Liposcelis bostrychophila, Sitophilus oryzae, Tenebrio molitor, Tribolium castaneum, Tribolium confusum*	[120,83,107,123]
p-cymene	*Lasioderma serricorne, Liposcelis bostrychophila, Tribolium castaneum, Trogoderma granarium*	[92,124]
Terpinen-4-ol	*Bemisia tabaci, Sitophilus granaries, Sitophilus oryzae, Sitophilus zeamais, Tenebrio molitor*	[81,107,119,125,126]
Thymol	*Bemisia tabaci, Lasioderma serricorne, Liposcelis bostrychophila, Plutella xylostella, Musca domestica, Reticulitermes dabieshanensis, Tribolium castaneum, Trogoderma granarium*	[86,92,93,97,124,126]
α-phellandrene	*Callosobruchus chinensis, Callosobrunchus maculatus*	[127]
α-pinene	*Callosobruchus chinensis, Callosobrunchus maculatus, Culex pipiens, Drosophila melanogaster, Lasioderma serricorne, Liposcelis bostrychophila, Sitophilus oryzae, Sitophilus zeamais, Thrips palmi, Tribolium castaneum, Tribolium confusum*	[120,83,84,90,123,127,128]
α-terpineol	*Bemisia tabaci, Callosobrunchus maculatus, Halyomorpha halys, Sitophilus granarius, Sitophilus oryzae, Sitophilus zeamais, Thrips palmi*	[81,90,100,112,125,126]
β-ionol	*Bemisia tabaci*	[129]
β-ionone	*Bemisia tabaci*	[129]
β-pinene	*Lasioderma serricorne, Thrips palmi, Tribolium castaneum*	[90,130]
γ-terpinene	*Callosobrunchus maculatus, Sitophilus oryzae, Tribolium confusum*	[83]
Monoterpenes as contact insecticides		
1,8-cineole	*Chrysomia megacephala, Helicoverpa armigera, Musca domestica, Pediculus humanus capitis, Poratrioza sinica*	[131,[132], [133], [134], [135]]
Camphor	*Anopheles stephensi, Aphis nerii, Musca domestica*	[131,89,136]
Carvacrol	*Aedes albopictus, Culex pipiens, Sitophilus zeamais, Tribolium castaneum, Trogoderma granarium*	[92,[137], [138], [139]]
Carvone	*Musca domestica, Poratrioza sinica, Sitophilus oryzae*	[131,135,140]
Citral	*Bemisia tabaci, Culex quinquefasciatus*	[102,141]
Citronellal	*Musca domestica, Drosophila suzukii, Sitophilus oryzae*	[131,104,135,140]
Cuminaldehyde	*Musca domestica, Poratrioza sinica, Sitophilus oryzae*	[131,140]
Eugenol	*Aedes albopictus, Blattella germanica, Musca domestica, Pediculus humanus capitis, Periplaneta americana*	[114,134,137,142]
Geranial	*Drosophila suzukii*	[104]
Geraniol	*Culex quinquefasciatus, Culex pipiens, Tribolium castaneum*	[102,143,144]
Guaiol	*Aedes albopictus*	[115]
Limonene	*Aedes albopictus, Culex pipiens molestus, Helicoverpa armigera, Poratrioza sinica, Sitophilus oryzae, Tribolium castaneum*	[109,135,[145], [146], [147]]
Linalool	*Aedes aegypti, Aedes albopictus, Chrysomia megacephala, Culex quinquefasciatus, Culex pipiens, Pediculus humanus capitis, Sitophilus oryzae*	[102,122,132,134,143,148]
Menthol	*Aphis nerii, Musca domestica*	[131,89]
Menthone	*Sitophilus oryzae*	[140]
Myrcene	*Aedes aegypti, Aedes albopictus, Culex quinquefasciatus, Lasioderma serricorne, Liposcelis bostrychophila, Myzus persicae, Tribolium castaneum*	[115,123,148,149]
p-cymene	*Culex quinquefasciatus, Lasioderma serricorne, Liposcelis bostrychophila, Musca domestica,* *Sitophilus oryzae, Tribolium castaneum, Trogoderma granarium*	[131,92,124,140,150,151]
Terpinen-4-ol	*Anopheles stephensi, Chrysomia megacephala, Helicoverpa armigera*	[132,133,136]
Thymol	*Aedes albopictus, Culex pipiens, Lasioderma serricorne, Liposcelis bostrychophila, Pediculus humanus capitis, Tribolium castaneum, Trogoderma granarium*	[92,95,124,134,137,139]
α-cedrol	*Culex quinquefasciatus*	[152]
α-phellandrene	*Culex pipiens, Lucilia cuprina*	[153,154]
α-pinene	*Aedes aegypti, Aedes albopictus, Culex pipiens, Culex quinquefasciatus, Incisitermes minor,Lasioderma serricorne, Liposcelis bostrychophila, Musca domestica, Poratrioza sinica, Rhyzopertha dominica, Sitophilus granaries, Sitophilus oryzae, Sitophilus zeamais, Tribolium castaneum, Tribolium confusum*	[131,123,135,137,140,152,[154], [155], [156], [157]]
α-terpinene	*Aedes albopictus, Musca domestica*	[131,137]
α-terpineol	*Aedes albopictus, Blattella germanica, Camponotus pennsylvanicus, Culex quinquefasciatus, Periplaneta americana*	[137,142,158]
β-pinene	*Aedes aegypti, Aedes albopictus, Incisitermes minor, Lasioderma serricorne, Mythimna separate, Poratrioza sinica, Semiaphis heraclei, Tribolium castaneum*	[130,135,137,156,157,159]
δ-3-Carene	*Culex pipiens, Culex quinquefasciatus*	[152,154]
γ-terpinene	*Aedes albopictus, Anopheles stephensi, Helicoverpa armigera*	[133,136,137]

Considering the exciting biological and beneficial activities of monoterpenes, this review discusses their various biological activities. These encompass insecticidal, repellent, antifeedant, plant defense, and antimicrobial effects against insect pests and their associated microbes.

## 3 Monoterpenes for plant defense

Monoterpenes play a crucial role in plant defense due to their diverse mechanisms of action. Primarily, they serve as direct deterrents to herbivores and pathogens [160]. Certain monoterpenes possess potent aromas and toxic characteristics, capable of repelling or incapacitating herbivores and inhibiting the proliferation of bacterial and fungal pathogens. For example, compounds such as limonene and menthol exhibit robust antimicrobial and insect-repellent attributes, establishing an instant chemical barrier against invading organisms [161].

Monoterpenes play vital roles in plants, fulfilling functions such as enhancing thermotolerance [162], acting as signaling molecules to bolster plant resilience against stressors like high temperatures [163], and contributing to antioxidant activities by scavenging free radicals and reducing metal ions such as Cu(II) and Fe(II) [164]. These compounds constitute essential constituents of essential oils, imparting aroma and flavor to both plants and processed foods [165]. Moreover, monoterpenes participate in regulating energy metabolism and exerting antimicrobial, analgesic, anti-inflammatory, antidiabetic, and anti-obesity effects [166]. However, it is crucial to recognize that while monoterpenes provide various benefits, some compounds may exhibit toxic effects under certain conditions. This underscores the need for careful evaluation of their potential impact on human health [167].

Monoterpenes play a crucial role in plant defense by acting as inducible volatiles that trigger innate immune responses. Research indicates that monoterpenes, such as geraniol, β-myrcene, and linalool, are synthesized in response to stress and pathogen infections, enhancing plant defense against pathogens [[168], [169], [170]]. These volatile compounds contribute to systemic acquired resistance (SAR), a broad-spectrum immune response in plants, by inducing the expression of defense-related genes and promoting resistance to pathogenic fungal infections [[171], [172], [173]]. Monoterpenes, like *α*-pinene and *β*-pinene, have been shown to propagate immunity within and between plants, strengthening SAR signaling networks and potentially fortifying crop protection strategies at a population level. Furthermore, the emission of monoterpenes from vegetation into the atmosphere can influence cloud formation and affect the Earth's radiative budget, underscoring the diverse ecological functions of these compounds [174,175].

Monoterpenes play a crucial role in plant defense by mediating interactions between plants and insects. They act as signaling molecules that attract predators or parasitoids of herbivores, supporting biological control strategies [25,176,177]. This tritrophic interaction displays the ecological efficiency of monoterpenes in plant defense. Additionally, monoterpenes contribute to allelopathy by inhibiting the growth of neighboring plants, thus securing essential resources. The biosynthesis of monoterpenes is regulated by environmental factors and plant development, demonstrating plant adaptability. Advances in molecular biology have unraveled the regulation of monoterpene synthesis, providing insights for sustainable pest management. Utilizing monoterpenes can reduce dependence on synthetic pesticides, promoting ecological balance and reducing environmental impact.

## 4 Monoterpenes: chemistry and biological activity

### 4.1 Chemical properties of monoterpenes

The chemical characteristics of monoterpenes are predominantly influenced by their diverse structures, encompassing acyclic, monocyclic, and bicyclic forms. This structural diversity profoundly impacts their reactivity, stability, and interactions with other chemical substances. Acyclic monoterpenes, like citronellal, myrcene and ocimene, exhibit an open-chain configuration, endowing them with considerable flexibility [178]. However, this openness also renders them more susceptible to oxidation and polymerization [[179], [180], [181]]. Monocyclic monoterpenes, such as limonene and menthol, possess a single-ring structure that enhances rigidity, thereby altering their chemical reactivity, particularly in reactions involving ring strain and steric hindrance [182,183]. Bicyclic monoterpenes, including pinene and camphene, feature two fused rings, introducing additional complexity to their chemical behavior due to constraints on molecular movement and the potential for unique stereoisomeric forms [184].

One of the distinctive chemical properties exhibited by monoterpenes is their susceptibility to electrophilic addition reactions, a characteristic attributed to the presence of double bonds within their molecular structure. These reactions play a pivotal role in the biosynthesis of more sophisticated terpenoids and in modifying monoterpenes in synthetic chemistry. Moreover, monoterpenes are subjected to oxidation, enzymatic and non-enzymatic, resulting in the generation of diverse oxygenated derivatives such as alcohols, aldehydes, ketones, and epoxides [185,186]. These derivatives frequently display heightened biological activity and serve as crucial intermediates in the manufacturing of flavors, fragrances, and pharmaceuticals.

Moreover, the stereochemistry of monoterpenes significantly influences their chemical behavior. The presence of chiral centers in numerous monoterpenes leads to the formation of multiple stereoisomers, each possessing unique physical and chemical properties. This phenomenon of stereoisomerism holds considerable importance not only in their biological functions but also in their sensory attributes, evident in the distinct aromas and flavors associated with different enantiomers of the same compound [187,188]. In natural ecosystems, monoterpenes fulfill diverse ecological roles, ranging from attracting pollinators to serving as defense mechanisms against herbivores and pathogens. Their volatile nature enables easy dispersion into the environment, facilitating their involvement in atmospheric reactions. Consequently, they contribute to the formation of secondary organic aerosols, thereby influencing air quality and climate dynamics.

### 4.2 Volatility and solubility

Understanding the volatility and solubility of monoterpenes is crucial for various scientific and industrial applications. Volatility, referring to a substance's tendency to vaporize, is a fundamental characteristic that shapes the aromatic profile and therapeutic effectiveness of monoterpenes. This trait is mainly determined by their molecular structure, intermolecular forces, and external factors such as temperature and pressure [189,190]. Monoterpenes, such as limonene, myrcene, and *α*-pinene, demonstrate high volatility, rendering them significant in the fragrance industry and influencing their roles in plant-insect interactions, where they function as attractants or repellents.

The distribution of monoterpenes in natural and artificial systems is determined by their solubility. This property hinges on both the polarity of the monoterpene molecule and the nature of the solvent. Generally, monoterpenes exhibit hydrophobic tendencies, resulting in low solubility in water but high solubility in organic solvents such as ethanol, acetone, and oils [191,192]. This characteristic plays a pivotal role in extraction and formulation processes, where the choice of solvent significantly impacts both yield and compound purity. For example, in pharmaceutical contexts, leveraging the solubility of monoterpenes in lipophilic environments enhances drug bioavailability.

Understanding the dynamic relationship between volatility and solubility holds crucial significance, not only in practical applications but also in unraveling the ecological significance of monoterpenes. These compounds, emitted by plants, play a pivotal role in atmospheric chemistry and biogeochemical cycles, thereby exerting influence on air quality and climate. Moreover, their solubility characteristics play a critical role in their absorption and transportation within plants, thereby exerting profound effects on plant physiology and defense mechanisms.

### 4.3 Biological activities

The diverse pharmacological properties and potential therapeutic applications of monoterpenes have sparked significant scientific interest in their biological activities. These activities primarily stem from their capacity to engage with a range of biological targets, including enzymes, receptors, and ion channels, resulting in a wide array of bioactive effects. Extensive research has revealed that monoterpenes (thymol, carvacrol, citral, *α*-pinene, *β*-myrcene, d-limonene, geraniol, linalool, cineole, as well as linalyl acetate) exhibit a spectrum of biological activities, encompassing anti-inflammatory, antimicrobial, antioxidant, anticancer, analgesic, and neuroprotective effects, positioning them as promising candidates for the development of innovative pharmaceutical agents [193,194].

Among the numerous biological activities of monoterpenes, their antimicrobial action stands out prominently. Monoterpenes, including thymol, carvacrol, and eucalyptol, demonstrate strong antibacterial, antifungal, and antiviral properties, which have been traditionally harnessed in medicine and are currently being investigated for their potential in addressing antibiotic-resistant pathogens [[195], [196], [197]]. These compounds disrupt microbial cell membranes, impede biofilm formation, and disrupt microbial metabolism, thereby thwarting growth and proliferation.

Another notable domain of study involves the anti-inflammatory and pain-relieving attributes of monoterpenes. Substances such as menthol and camphor are extensively employed in topical pain-relief products for their refreshing and calming effects [198,199]. Monoterpenes (*γ*-Terpinene, *α*-terpineol, *α*-carveol, menthone and pulegone) regulate inflammatory processes by blocking essential enzymes like cyclooxygenase and lipoxygenase, thus diminishing the generation of inflammatory agents [200,201]. This renders them beneficial in managing inflammatory ailments like arthritis and muscle discomfort.

In the field of cancer treatment, certain monoterpenes, including limonene and perillyl alcohol, exhibit promising anti-cancer properties [202]. These compounds function by triggering apoptosis, restraining cell proliferation, and adjusting signaling pathways crucial in the advancement of cancer [203]. Effectiveness of monoterpenes (D-limonene, perillyl alcohol, perillic acid and limonene 1, 2-diol) has been scrutinized across various cancer types, including breast, lung, and liver cancers, underscoring the potential of monoterpenes as supplementary therapies in oncology. Additionally, monoterpenes show promise in protecting neural cells [[204], [205], [206]]. Compounds like carvacrol, citronellal, linalool and geraniol have displayed potential in mitigating neurodegenerative conditions like Alzheimer's and Parkinson's diseases [207,208]. Their mechanisms of action involve antioxidant properties, inhibition of acetylcholinesterase, and modulation of neurotransmitter systems, collectively contributing to the safeguarding of neuronal cells and the enhancement of cognitive functions [209].

Monoterpenes, besides their pharmacological uses, show great potential in agriculture and food preservation. Their innate insecticidal and fungicidal qualities provide eco-friendly substitutes for synthetic pesticides, promoting sustainable agricultural methods. Furthermore, their antioxidant characteristics play a crucial role in prolonging the shelf life of food items, thus diminishing food spoilage and waste.

### 4.4 Antimicrobial properties

The historical utilization of plant extracts and essential oils in traditional medicine highlights the enduring acknowledgment of the antimicrobial effectiveness of monoterpenes. Recent scientific investigations have shed light on the mechanisms through which monoterpenes exert their antimicrobial properties, thus enhancing our understanding of their potential applications. Compounds, like ascaridol, carvone, limonene, menthol, methyl eugenol, thymol, toosendanin and volkensin, classified as monoterpenes, have displayed broad-spectrum antimicrobial capabilities against diverse pathogens encompassing bacteria, fungi, and viruses [39,194].

The actions of monoterpenes are complex, encompassing the disruption of microbial cell membranes, interference with intracellular processes, and the inhibition of biofilm formation. These characteristics render monoterpenes highly promising as potential substitutes or supplements to traditional antimicrobial agents, which are facing growing challenges from resistant microbial strains. The hydrophobic properties of monoterpenes enable them to incorporate into the lipid bilayers of microbial membranes, inducing disruptions in both structure and function, ultimately resulting in cell death [[210], [211], [212]].

Furthermore, the combined use of monoterpenes with other antimicrobial agents presents new opportunities to enhance the efficacy of existing treatments. This collaboration could decrease the necessary dosage of traditional antibiotics, thereby alleviating adverse effects and impeding the advancement of resistance. The investigation into monoterpenes goes beyond their direct antimicrobial attributes. Their capacity to regulate the immune response, along with their potential as anti-inflammatory and antioxidant agents, contributes to a comprehensive strategy for addressing infections. Moreover, the biocompatibility and relatively low toxicity of monoterpenes bolster their suitability for therapeutic applications.

## 5 Monoterpenes for insect pest management

**Fig. 1:**
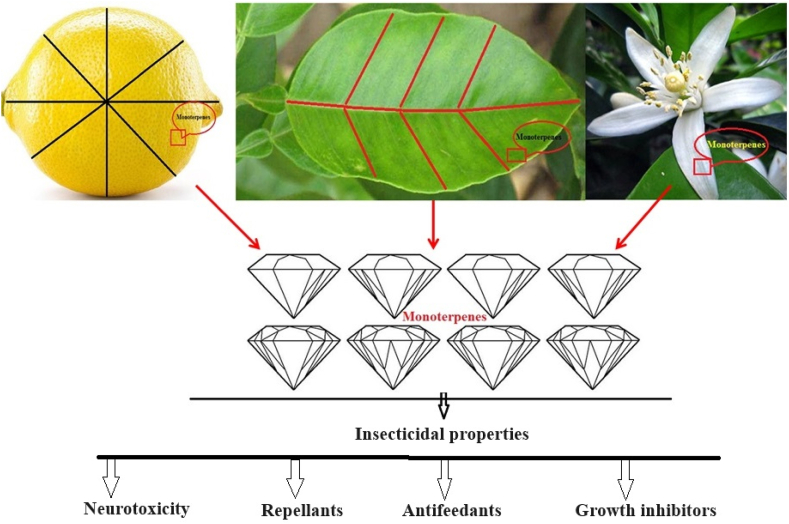
Diagrammatic sketch of monoterpenes with insecticidal properties.

For decades, insects have presented substantial challenges to agriculture, public health, and food preservation, initiating an ongoing quest for effective pest management strategies [[213], [214], [215]]. While conventional synthetic insecticides have proven effective, they frequently result in harmful environmental impacts, the emergence of insect resistance, and potential threats to human health. Consequently, there is an increasing fascination with investigating alternative, environmentally friendly insecticidal solutions. A particularly promising area of study involves examining monoterpenes, a varied group of naturally derived organic compounds [131,216]. Monoterpenes are effective insect pest, by various pathways, like direct toxicity, insect repellant, insect antifeedant, and growth inhibitors (Fig. 1).

The insecticidal potential of monoterpenes (carvone, (−)-borneol, (+)-camphor, 1,8-cineole, cuminaldehyde, eucalyptol and linalool) displays by their diverse modes of action, and function as neurotoxins, disrupting the nervous systems of insects, or as fumigants, inducing respiratory distress [217,87]. Moreover, certain monoterpenes possess repellent properties, discouraging insects from approaching treated areas. The effectiveness of these compounds varies based on the insect species, the specific monoterpene utilized, and its concentration. The appeal of monoterpenes as insecticides lies not only in their efficacy but also in their relative safety for non-target organisms, including humans. Being natural compounds, they tend to degrade more swiftly in the environment, thereby mitigating the risk of long-term ecological harm [218,219]. This aligns with the global movement towards sustainable agricultural practices and integrated pest management (IPM) strategies, which prioritize environmental health and safety.

Furthermore, incorporating monoterpenes in agriculture could improve economic viability by decreasing reliance on synthetic pesticides and potentially reducing production costs. This is especially significant for organic farming, where synthetic chemicals are restricted. In these systems, monoterpenes could play a vital role in pest management programs, aiding in crop protection and helping to meet organic certification standards. Despite their potential, using monoterpenes as insecticides presents several challenges. Problems like high volatility, rapid degradation, and inconsistent effectiveness in different environmental conditions require continuous research to improve formulations and delivery methods. Advances in nanotechnology and encapsulation techniques are being investigated to increase the stability and efficacy of monoterpene-based insecticides [220,221].

Synthetic insecticides are employed globally to control agricultural and public health insect pests. The use of conventional insecticides is considered a primary factor in the significant increase in agricultural productivity over the past seven decades [222]. However, the prolonged and widespread use of these chemicals has raised public concerns regarding their impact on human health and the environment. The unwise use of synthetic pesticides has led to several issues, including food contamination from pesticide residues, environmental pollution, health hazards, the development of pest resistance, and adverse effects on natural enemies and other non-target organisms [223,224]. Consequently, there is a global movement towards reducing the use of synthetic chemicals and finding alternative pest control strategies that pose minimal or no risk to humans and the environment [225,226]. As a result, many countries have recently implemented IPM programs. The development of new biopesticides is considered one of the most promising tools for pest management within the IPM framework. It is projected that the global biopesticide market will grow by up to 20 % by 2025 [227].

Plant-based products such as powders, extracts, essential oils, and pure secondary metabolites serve as the active ingredients in botanical insecticides, a primary category of biopesticides. These botanical insecticides offer several advantages over traditional synthetic insecticides, including high biodegradability, rapid action, novel mechanisms of action, low toxicity to mammals, selectivity, and minimal impact on plants [167,222,228]. These characteristics contribute to resolving the challenges linked to the use of synthetic insecticides. In fact, plant-derived natural products have proven to be effective antifeedants, repellents, and toxicants against economically significant insects in agriculture and medicine [216,228,229]. Monoterpenes and essential oils are among the most important plant metabolites, and they are considered valuable alternatives for insect control [228,230].

Monoterpenes are common secondary metabolites in plants, but they are also found in other organisms, including microbes and insects [231]. They contribute significantly to the aroma of plants and are the primary odoriferous compounds in many flowers and fruits. Monoterpenes are typically isolated as major constituents of essential oils from aromatic plants through hydrodistillation or solvent extraction. These lipophilic liquids are highly volatile [232]. They provide protection for plants against external abiotic factors like heat, water loss, and UV radiation [25,233], as well as biotic threats such as parasites, pathogens, and herbivores [25,35]. Additionally, monoterpenes play a crucial role in attracting pollinators and seed dispersers, facilitating plant-to-plant signaling, and engaging in allelopathy [63,234].

Among the various plant secondary metabolites employed for insect control over the decades, monoterpenes stand out as one of the most effective groups of botanical insecticides [223]. Monoterpenes are considered excellent alternatives to conventional synthetic insecticides for several reasons. Many monoterpenes demonstrate a wide range of biological activities against both agricultural and public health pests. These activities include insecticidal [217,235], antifeedant [236,237], repellent [[238], [239], [240]], and insect growth regulatory properties [236,241].

In agriculture, monoterpenes are effective in controlling post-harvest diseases in vegetables and fruits [242]. Essential oils, which contain monoterpene mixtures and individual monoterpenes, are frequently used as flavoring agents. Notable examples of monoterpenes in the flavor and fragrance industries include menthol (*Mentha arvensis*), d-carvone (*Carum carvi*), d-limonene (*Citrus sinensis*), citral (*Cymbopogon citratus*), and 1,8-cineole from *Eucalyptus globulus* [243].

Monoterpenes constitute a significant class of secondary metabolites found in aromatic and medicinal plants. Over 1000 monoterpenes have been isolated and identified from various sources [244]. Monoterpenes offer several advantages over traditional synthetic insecticides, including high biodegradability, low toxicity to mammals, selectivity, and minimal impact on plants, non-target organisms, and the environment [228]. These attributes are crucial in addressing the issues associated with synthetic insecticide use. The effectiveness of monoterpenes as protectants for stored grain has been investigated against numerous stored product insects [245,246]. Saad and Abdelgaleil [247] specifically tested the insecticidal efficacy of various monoterpenes against stored grain insect pests, finding that some monoterpenes provided satisfactory management.

### 5.1 Repellent properties

Insects play essential roles in ecosystems and agriculture but also present significant challenges to human health. They are vectors for diseases like malaria, dengue fever, and Lyme disease, and cause substantial agricultural damage as pests [248,249]. This highlights the need for effective insect repellents. Traditionally, chemical repellents such as DEET (N,N-Diethyl-meta-toluamide) have been widely used [250]. However, concerns about their environmental impact and potential health risks have urged the search for safer and natural alternatives. Monoterpenes, naturally occurring compounds found mainly in the essential oils of various plants, are among the promising candidates. Insects heavily depend on chemical cues for survival and reproduction, using them for foraging, mating, and evading predators [251,252]. Their olfactory system is highly sensitive and adapted to detect a broad range of chemical signals, including monoterpenes [253]. These compounds could influence insect behavior by attracting, repelling, or disrupting their normal physiological processes.

The effectiveness of monoterpenes as insect repellents is attributed to their volatility, allowing them to evaporate quickly and create a protective barrier against insect attacks [238]. These compounds disrupt the olfactory receptors of insects, hindering their ability to locate hosts or food sources. The repellent action of monoterpenes involves complex molecular interactions that influence the behavior and physiology of target insects [71,254]. Research into the insect-repellent properties of monoterpenes encompasses various disciplines, including organic chemistry, entomology, and environmental science. Studies have investigated the effectiveness of different monoterpenes against numerous insect species, the optimal concentrations for repellent activity, and the potential for developing commercial products based on these natural compounds [58,238,239]. Moreover, the environmental advantages of using monoterpenes, which are biodegradable and pose minimal risks to non-target organisms, further support their use in IPM strategies.

The study of monoterpenes displays a crucial basis for recognizing their importance and potential as insect repellents. By exploring the chemical properties, mechanisms of action, and practical applications of these compounds, we can better understand their role in creating sustainable and effective insect control solutions. Ongoing research into the diverse functions of monoterpenes highlights their promise in improving pest management practices, thereby benefiting public health and promoting environmental conservation.

### 5.2 Growth inhibition and developmental disruption

Certain monoterpenes inhibit the growth and development of insect pests [241,255]. Research has shown that compounds like eucalyptol, S-(+)-carvone, trans-anethole and thymol disrupt the molting process and impede larval growth, thereby reducing the reproductive success of pest populations [256]. Monoterpenes affect insect growth through multiple pathways. They may interfere with the neuroendocrine system, which is crucial for regulating insect growth and development. Monoterpenes have been found to impact the juvenile hormone and ecdysone pathways, essential for molting and metamorphosis [[257], [258], [259]]. Additionally, monoterpenes act as antifeedants, diminishing nutrient intake and the energy resources available for growth [60]. Another significant mechanism is enzyme inhibition, where monoterpenes block key digestive enzymes, impairing nutrient absorption and utilization.

The developmental disruptions caused by monoterpenes result in various physiological and morphological effects. Exposure to these compounds leads to abnormalities during molting, delayed pupation and emergence, and in severe cases, mortality at the larval or pupal stages [260]. Certain monoterpenes cause morphological deformities and obstruct the successful transition from larval to adult stages [255]. These disruptions are attributed to the compounds' interference with hormonal regulation and their direct toxic effects on developing tissues.

Understanding the effects of monoterpenes on insect growth and development holds significant ecological and practical importance. Ecologically, these compounds play a role in naturally regulating insect populations and shaping plant-insect interactions [261]. Practically, using monoterpenes or monoterpene-rich essential oils as biopesticides offers an eco-friendly alternative to conventional chemical pesticides [262,263]. This approach provides a sustainable method of pest management by minimizing the risk of resistance and reducing harm to non-target organisms and ecosystems. Despite the promising potential of monoterpenes for insect control, several challenges remain. The efficacy of monoterpenes vary due to differences in their composition across plant species and environmental conditions [264]. Additionally, developing delivery systems that maintain effective concentrations at target sites without causing phytotoxic effects is essential. Future research should focus on understanding the synergistic effects of monoterpenes with other plant secondary metabolites and their impacts on a broader range of insect species. Furthermore, incorporating monoterpenes into IPM programs requires comprehensive studies on their long-term ecological impacts and cost-effectiveness.

### 5.3 Direct toxicity to insects

Insects are crucial to ecosystems, contributing to pollination, decomposition, and serving as vital food sources for other animals. However, certain insect species are notorious pests, causing significant damage to agriculture, stored products, and even human health. Although traditional insecticides are effective, they often pose problems such as environmental toxicity, harm to non-target species, and the development of insect resistance [[265], [266], [267]]. These issues highlight the need to explore alternative insect control strategies that are both effective and sustainable. The importance of monoterpenes in insect neurotoxicity is emphasized by their widespread occurrence in nature and their diverse modes of action [166]. Essential oils rich in monoterpenes, such as those from eucalyptus, lavender, and peppermint, have been used historically as insect repellents and pesticides [268,269]. The growing demand for eco-friendly and sustainable pest control solutions has increased scientific interest in understanding the neurotoxic effects of monoterpenes on insects.

Monoterpenes exert neurotoxic effects on insects through various mechanisms. These compounds disrupt the insect nervous system by modulating ion channels, receptors, and enzymes essential for nerve transmission. Some monoterpenes interfere with gamma-aminobutyric acid (GABA) receptors, causing uncontrolled neural activity that leads to paralysis or death [77,270]. Others inhibit acetylcholinesterase, an enzyme vital for breaking down acetylcholine, which results in the accumulation of this neurotransmitter and continuous stimulation of muscles and nerves [65,271]. Monoterpenes, including limonene, α-pinene, camphor, and menthol, have demonstrated potential in disrupting insect physiology through various cytotoxic effects. These effects may range from neurotoxicity, which causes paralysis and death, to the disruption of cell membranes and metabolic processes. The cytotoxic effects of monoterpenes in insects involve complex mechanisms, which could inhibit acetylcholinesterase, resulting in the buildup of acetylcholine, which in turn causes excessive neuronal activity [65,272]. Additionally, monoterpenes induce oxidative stress by generating reactive oxygen species (ROS), which damage cellular components such as lipids, proteins, and DNA [[120], [273], [274]]. Some monoterpenes may also interfere with the respiratory system of insects by disrupting mitochondrial function, resulting in energy depletion and cell death.

Using monoterpenes as insecticides offers an environmentally friendly alternative to synthetic chemicals. These compounds are biodegradable and generally less toxic to non-target organisms, including humans and beneficial insects such as pollinators and natural predators [60,218,265]. Additionally, plants naturally produce monoterpenes as part of their defense mechanisms against herbivores and pathogens, suggesting a lower likelihood of resistance development compared to synthetic insecticides [25,65,169]. Developing monoterpene-based insecticides involves extensive research into their efficacy, formulation, and application methods. Studies focus on identifying the most potent monoterpenes, understanding their specific modes of action, and optimizing delivery systems to enhance their stability and effectiveness under various environmental conditions [216,235]. Furthermore, research into the synergistic effects of combining different monoterpenes or integrating them with other biopesticides can lead to more robust pest management strategies.

## 6 Monoterpenes for the pathogenesis of entomopathogenic fungi

Monoterpenes, VOCs abundant in plants, have recently attracted considerable interest for their potential to alter the pathogenesis of entomopathogenic fungi (EPF) targeting insect pests. These compounds serve multiple roles in plant defense, such as repelling insect herbivores and attracting their predators [275,276]. Recent studies have revealed their complex involvement in modulating interactions between plants, insects, and fungi [277,278]. Therefore, understanding the impact of monoterpenes on the EPF pathogenesis is essential for developing innovative strategies in IPM and sustainable agriculture. This comprehensive review will explore the mechanisms by which monoterpenes influence fungal pathogenicity, their potential synergistic or antagonistic effects, and their implications for enhancing biological control strategies against insect pests.

Effective management of insect pests is crucial in agriculture, forestry, and public health, requiring strategies that are both effective and sustainable. Although traditional chemical insecticides are effective, they often result in environmental pollution, the development of resistance, and adverse effects on non-target species. As a result, biological control agents, especially EPF, have gained significant attention due to their environmentally friendly and target-specific action against various insect pests. EPF, like *Beauveria bassiana, Metarhizium anisopliae,* and *Cordyceps fumosorosea*, infect and kill insects through a process involving spore adhesion, germination, and penetration of the host cuticle, followed by internal proliferation and toxin production [279,280]. However, the efficacy of EPFs could be affected by various factors, including environmental conditions, host immunity, and the presence of chemical compounds like monoterpenes.

EPF offer a promising alternative to chemical insecticides, but their effectiveness in the field vary due to environmental factors such as temperature, humidity, and UV exposure. Furthermore, their pathogenicity is greatly influenced by interactions with other biotic and abiotic elements [281,282]. The relationship between monoterpenes and EPF is complex and could significantly affect how these fungi combat insect pests. Monoterpenes have the ability to modify the physiology and immune responses of insect hosts [71,283], thereby either increasing or decreasing their susceptibility to fungal infections. Additionally, the volatile nature of monoterpenes can create microenvironments that either aid or impede the dispersal and persistence of fungal spores.

Recent studies have explored the synergistic impacts of monoterpenes on the EPF pathogenicity against insect pests. Synergism arises when the combined effect of two agents surpasses the sum of their individual effects [284,285]. In the realm of pest management, this implies that the concurrent application of monoterpenes and EPF could reinforce the overall effectiveness of pest control strategies. Monoterpenes may augment fungal infection by compromising the insect's immune system, disrupting the insect cuticle to facilitate fungal penetration, or by serving as attractants that boost contact rates between the fungi and their insect hosts.

The effectiveness of EPF as biocontrol agents may vary due to several factors, such as environmental conditions and interactions with other compounds [286,287]. Recently, monoterpenes have emerged as a noteworthy group of compounds in pest management. Their synergistic effects on the EPF pathogenicity against insect pests offer a promising avenue for enhancing biocontrol efficacy. Synergy, in this context, refers to the phenomenon where the combined impact of monoterpenes and EPF exceeds the sum of their individual effects [285,288]. This synergistic interaction can manifest in various ways, including heightened fungal virulence, enhanced spore germination, improved infection of insect hosts, and faster insect mortality.

Understanding the mechanisms driving this synergy is essential for maximizing the efficacy of monoterpenes and EPF in IPM programs. Studies have shown that monoterpenes alter the physiology and immune reactions of insect pests, rendering them more vulnerable to fungal infections [71,283]. Furthermore, specific monoterpenes might directly boost fungal proliferation and pathogenicity by modulating fungal metabolism and stress response pathways. These revelations facilitate to develop biopesticides that combine the advantages of EPF and monoterpenes, potentially leading to more potent formulations.

Furthermore, the utilization of monoterpenes with EPF is in accordance with the principles of sustainable agriculture. This strategy harnesses natural compounds and organisms, thereby mitigating the environmental impact of pest control methods and diminishing dependence on synthetic chemicals. With the increasing demand for organic and sustainable agricultural goods, the advancement of these integrated biocontrol tactics gains significant relevance.

## 7 Impact of monoterpenes on insect-associated microbes

Insects, comprising one of the most expansive and diverse organism groups, frequently establish complex relationships with microbes. These microorganisms either serve beneficial roles, facilitating functions like digestion, nutrient absorption, and immune response [289,290], or pose a threat as pathogens, triggering diseases that profoundly affect insect survival and reproduction [291]. The symbiotic bonds forged between insects and microbes play a pivotal role in the ecological triumph and adaptability of numerous insect species.

Research into the relationship between monoterpenes and microbes associated with insects is rapidly expanding, shedding light on the diverse functions these compounds serve in shaping microbial communities within insect hosts [276,292,293]. Monoterpenes can exert a range of effects on these microbes, from inhibiting pathogenic bacteria and fungi to potentially disrupting beneficial symbiotic relationships. Thorough understanding of these interactions is crucial for deciphering the complex dynamics between insects and microbes and understanding their broader ecological significance.

Monoterpenes have the capacity to directly impact microbial viability by penetrating cell membranes and interfering with metabolic processes, ultimately resulting in microbial cell demise. Furthermore, they possess the potential to shape the structure and functionality of microbial communities residing within insects, potentially modifying the insects' well-being and behavioral patterns [276]. Thus, monoterpenes could jeopardize the stability of gut microbiota in insects, thereby impacting their digestive efficiency and ability to absorb nutrients. Conversely, certain insects have developed mechanisms to endure or exploit monoterpenes [294,295], assimilating them into their own chemical defenses or utilizing them as signals for locating hosts and mates.

Research about the monoterpene effects on insect-associated microbes covers multiple disciplines, including entomology, microbiology, ecology, and chemical ecology. Investigations have explored these interactions across diverse scenarios, notably in pest management, where monoterpenes are scrutinized for their capacity to regulate insect populations by acting upon their microbial symbionts [296,297]. Furthermore, understanding the ecological function of monoterpenes in shaping insect-microbe associations offers valuable insights into natural pest resistance mechanisms in plants and the evolutionary forces that drive these interactions.

The gut microbiota of insects plays a crucial role in their overall health and functioning. These microbial communities are indispensable for tasks like digestion, nutrient absorption, detoxification of harmful substances, and defense against pathogens. They contribute significantly to the development of the host's immune system and overall physiological balance. The composition of these microbial communities is shaped by various factors, including diet, environment, and host genetics [298,299]. The interaction between monoterpenes and insect gut microbiota is a burgeoning field of research with substantial ecological, agricultural, and biotechnological implications. Monoterpenes, as potent bioactive compounds, can impact the gut microbiota in diverse ways. These effects may be advantageous or detrimental, contingent on the concentration and specific type of monoterpene, as well as the particular microbial species present in the gut.

Monoterpenes influence the metabolic activities of gut microbiota. Some are metabolized by these microbes, leading to the production of metabolites that exert various effects on the host insect, ranging from facilitating detoxification processes to modulating immune responses [293,300]. Changes in the composition and function of gut microbiota due to monoterpene exposure subsequently affect insect physiology and behavior. These changes may encompass alterations in nutrient absorption efficiency, growth rates, reproductive success, and even shifts in feeding behavior and habitat preference [301,302]. Understanding the impact of monoterpenes on insect gut microbiota holds profound ecological and agricultural significance. In natural ecosystems, these interactions shape plant-insect dynamics, thereby influencing plant health and insect population structures. In agricultural settings, manipulating the effects of monoterpenes could present novel strategies for pest management by manipulating gut microbiota to regulate pest populations [303]. Moreover, insights into these interactions could facilitate the development of probiotics or other microbiota-targeted interventions to improve the health and productivity of beneficial insects, such as pollinators and biocontrol agents.

Microorganisms play vital roles in an insect's ecosystem by aiding in digestion, acquiring nutrients, detoxifying harmful substances, and avoiding pathogens. Interrupting this complex microbial balance can significantly impact the health of insects, affecting factors such as its fitness, reproductive success, and susceptibility to diseases. Monoterpenes, with their antimicrobial properties, selectively target and suppress specific microbial populations in the insect gut [197]. This disruption can trigger dysbiosis, an imbalance in the microbiome, impairing the insect's ability to efficiently process food, weakening its immune system, and compromising its overall health. Notably, research indicates that exposure to certain monoterpenes decrease the presence of beneficial gut bacteria in insects like honeybees, potentially heightening their vulnerability to diseases and reducing their foraging efficiency [[304], [305], [306]]. Likewise, in beetles, monoterpenes have been observed to interfere with essential microbial symbionts responsible for detoxifying plant defenses [307,308], thereby impacting the insect's survival and reproduction.

## 8 Environmental and health considerations

Monoterpenes play a critical role in atmospheric chemistry, being abundantly emitted by vegetation and contributing significantly to the SOAs creation through atmospheric oxidation processes [51]. These aerosols exert influence on cloud formation and climate dynamics by modulating the Earth's radiative balance [55,56]. Monoterpenes undergo reactions with various atmospheric oxidants, such as hydroxyl radicals, ozone, and nitrate radicals, yielding diverse reaction products that either facilitate or attenuate aerosol formation [[52], [53], [54]]. This dual functionality underscores the crucial importance of monoterpenes for knowing and accurately modeling climate change dynamics. Furthermore, monoterpenes participate in the VOC emission, exerting substantial impacts on air quality. Their interactions with anthropogenic pollutants stimulate the formation of tropospheric ozone, a significant constituent of urban smog [309]. The regulatory implications of these emissions are profound, necessitating comprehensive studies to guide air quality management and environmental policy formulation.

The exploration of monoterpenes and their impact on non-target species has attracted considerable attention in recent years, especially with the increasing use of natural products in pest management and other industrial domains. Monoterpenes represent VOCs predominantly present in essential oils derived from various plants, including conifers, citrus fruits, and herbs such as mint and thyme. These compounds serve pivotal roles in plant defense mechanisms, the attraction of pollinators, and the deterrence of herbivores [25,60,234]. Nevertheless, their extensive application and environmental dissemination raise significant concerns regarding their effects on non-target species, encompassing beneficial insects, aquatic organisms, and terrestrial wildlife [176,310,311]. In agriculture, they are utilized as biopesticides, presenting a natural alternative to synthetic chemicals. While these compounds are praised for their reduced toxicity to humans and their potential to alleviate the adverse impacts of synthetic pesticides, their ecological safety remains incompletely understood. An expanding research suggests that monoterpenes can yield diverse and occasionally adverse effects on non-target species, inspiring investigations for their ecological compatibility and safety.

The influence of monoterpenes on non-target species is complex. For beneficial insects such as pollinators and natural pest predators, exposure to monoterpenes could disrupt various aspects of their behavior, reproduction, and survival rates. Research indicates that monoterpenes hinder their learning and memory, essential for efficient foraging [304]. Likewise, natural predators like ladybugs and predatory mites, instrumental in managing pest populations, may also suffer adverse effects, potentially causing unintended ecological disruptions.

Aquatic ecosystems are susceptible to the impacts of monoterpenes as well. These substances can infiltrate water sources via agricultural runoff and industrial discharges, potentially harming fish and invertebrate populations. Studies have shown that specific monoterpenes could be harmful to fish, causing respiratory problems and disrupting the delicate balance of aquatic food chains [312]. Furthermore, monoterpenes have the potential to build up in aquatic organisms over time, presenting lasting dangers to both wildlife and humans who consume them. Terrestrial wildlife, including birds and mammals, may also face risks from monoterpenes. These compounds enter terrestrial environments through air and soil pollution, affecting the health and behaviors of various species [[313], [314], [315]]. Birds, which often depend on scent for communication and foraging, may exhibit altered behaviors that jeopardize their survival and reproductive success. Mammals exposed to monoterpenes through inhalation or ingestion may display a range of toxic effects, from mild irritation to severe organ damage, depending on the concentration and duration of exposure.

### 8.1 Health considerations

From a health perspective, monoterpenes exhibit both advantageous and detrimental effects. Many monoterpenes are renowned for their therapeutic characteristics [40]. For instance, limonene, present in citrus peels, has displayed anti-inflammatory, antioxidant, and anticancer properties [316,317]. Likewise, *α*-pinene carries anti-inflammatory and bronchodilator qualities, rendering it valuable in both traditional and contemporary medicinal applications [318,319]. Conversely, exposure to elevated concentrations of monoterpenes, particularly in occupational environments like the flavor, fragrance, and cleaning industries, present health hazards [167]. Inhaling or coming into contact with certain monoterpenes could result in respiratory irritation, skin sensitization, and other adverse health outcomes. Moreover, the oxidation byproducts of monoterpenes, generated through reactions with indoor ozone, could exhibit greater toxicity than the original compounds, prompting concerns about indoor air quality.

The safety of monoterpenes for both humans and animals has sparked considerable interest and concern. With these compounds finding their way into a growing number of everyday products, it's imperative to grasp their potential health implications. While monoterpenes like limonene, pinene, and menthol are generally regarded as safe under specific usage conditions, their impacts can fluctuate significantly based on factors such as concentration, exposure route, and individual susceptibility.

### 8.2 Human safety considerations

Monoterpenes present various health hazards to humans, especially when exposure levels are elevated or individuals have underlying health issues. Common pathways of exposure include inhalation, dermal contact, and ingestion. Taking high concentrations of monoterpenes could cause respiratory irritation and, in severe instances, neurotoxic effects. Similarly, dermal contact may lead to allergic reactions or skin sensitization, particularly among those with heigh sensitivity. Consumption of products with high monoterpenes could induce gastrointestinal disturbances. Despite these risks, monoterpenes also confer numerous health advantages, including antimicrobial, anti-inflammatory, and anticancer properties [207]. Notably, menthol is widely recognized for its analgesic and cooling properties, whereas limonene shows promise in alleviating anxiety and stress. Consequently, realizing the safety profile of monoterpenes requires a sophisticated understanding of both their therapeutic benefits and potential hazards.

### 8.3 Animal safety considerations

Across animals, the effects of monoterpenes vary significantly and are influenced by factors such as size, metabolic rate, and habitat. Domesticated animals and wildlife may encounter monoterpenes through environmental sources, feed, and veterinary products. While some monoterpenes serve as beneficial natural pest repellents or components of medical treatments, others could be toxic if ingested or inhaled in large quantities. Research indicates that certain monoterpenes induce adverse effects in animals, including neurological disturbances, reproductive toxicity, and liver damage. For instance, d-limonene, commonly utilized as a flea and tick repellent, has been linked to adverse effects in cats due to their unique metabolic pathways. Similarly, excessive exposure to monoterpenes in livestock result in reduced feed intake and impaired growth performance [226,320,321].

## 9 Future directions and research needs

### 9.1 Genetic engineering for enhanced production of monoterpenes

Although monoterpenes play a crucial role, their extraction from natural sources poses numerous challenges. Conventional techniques like steam distillation and solvent extraction frequently prove inefficient, resulting in low yields and environmental repercussions [322,323]. Additionally, the inherent low production of monoterpenes in plants, coupled with fluctuations due to environmental and genetic factors, impedes consistent and scalable production required to fulfill industrial requirements.

### 9.2 Role of genetic engineering

Genetic engineering offers a promising pathway for addressing these challenges by facilitating the enhanced production of monoterpenes through a range of biotechnological interventions. Progress in synthetic biology and metabolic engineering enables precise manipulation of metabolic pathways in both microorganisms and plants, thereby optimizing them to yield higher quantities of monoterpenes [324,325].

Through the identification and manipulation of key enzymes involved in the biosynthesis of monoterpenes, scientists have the ability to augment the flow towards desired compounds. For example, the upregulation of genes responsible for monoterpene synthases or the engineering of preceding pathways to augment the provision of precursors can substantially elevate production. Microorganisms, such as *Escherichia coli* and *Saccharomyces cerevisiae*, could be genetically engineered to express genes related to plant monoterpene biosynthesis [324,326]. These hosts are often more receptive to genetic modifications and could be cultivated under controlled conditions, offering a stable and scalable platform for monoterpene production. Contemporary gene-editing techniques like CRISPR-Cas9 enable precise modifications within plant genomes [327], thereby enhancing monoterpene production through the elimination of competing pathways or the introduction of novel biosynthetic capabilities.

The refinement of monoterpene production systems can be advanced through the development of synthetic pathways and the optimization of regulatory networks. By crafting synthetic gene circuits, scientists can dynamically control metabolic fluxes, resulting in increased yields and enhanced efficiency.

### 9.3 Discovery of new monoterpenes with potent activities

The identification of novel monoterpenes boasting potent biological activities stands as a pivotal frontier in both natural product chemistry and pharmacology. This exploration of monoterpenes has gained considerable traction, driven by advancements in analytical techniques such as high-performance liquid chromatography (HPLC), gas chromatography-mass spectrometry (GC-MS), and nuclear magnetic resonance (NMR) spectroscopy [328,329]. These cutting-edge technologies empower precise identification and characterization of newfound monoterpenes sourced from various natural reservoirs including plants, fungi, and marine organisms. Moreover, the fusion of bioinformatics and cheminformatics has streamlined the prediction and modeling of monoterpene biosynthesis [330], thereby augmenting the discovery endeavor.

Recent research has shed light on the potential of monoterpenes in tackling the challenge posed by multidrug-resistant pathogens, which is increasingly becoming a global health concern [331]. Notably, compounds such as thymol and carvacrol have exhibited significant antibacterial properties against resistant strains, emphasizing the therapeutic potential of monoterpenes. Furthermore, monoterpenes like limonene and perillyl alcohol are currently under investigation for their potential in combating cancer, displaying promising efficacy in preclinical models of various cancer types through mechanisms such as apoptosis induction and cell cycle arrest [202]. The environmentally sustainable sourcing of monoterpenes is also a crucial aspect driving research efforts. Sustainable extraction techniques, biotechnological methods such as metabolic engineering in microbial hosts, and synthetic biology approaches are being employed to boost both the yield and variety of monoterpenes without causing depletion of natural resources. This aligns with the broader objectives of green chemistry and sustainable development. Additionally, the synergistic interactions between monoterpenes and existing pharmaceuticals offer new opportunities for combination therapies, potentially enhancing treatment effectiveness while reducing side effects associated with conventional treatments. Furthermore, the exploration of monoterpene derivatives and analogs expands the chemical landscape, providing a wide array of compounds for pharmacological evaluation and development.

### 9.4 Use of monoterpenes in organic farming

Organic farming has emerged as a sustainable alternative to conventional agricultural practices, emphasizing environmental health, biodiversity preservation, and human well-being. Within this framework, the use of monoterpenes has attracted considerable attention as a natural and environmentally friendly solution for various agricultural challenges. Monoterpenes, a class of organic compounds abundant in plant essential oils, possess diverse biological activities that render them invaluable in organic farming practices [332,333]. This comprehensive introduction aims to investigate the multifaceted role of monoterpenes in organic farming, exploring their chemical properties, mechanisms of action, and practical applications across diverse agricultural contexts. From pest management to soil health enhancement and beyond, the incorporation of monoterpenes offers a promising pathway for advancing sustainable agricultural systems that prioritize ecological balance and long-term viability.

Monoterpenes are essential in nurturing soil health and augmenting plant growth within organic farming systems. Their antimicrobial properties enable them to curb soil-borne pathogens like fungi and nematodes, thus lessening disease occurrence and fostering robust root systems [334,335]. Moreover, specific monoterpenes display allelopathic effects that can bolster seed germination, enhance root growth, and elevate overall crop yield.

### 9.5 Monoterpenes for urban pest control

Urban pest control plays a vital role in safeguarding public health and upholding the well-being of urban landscapes. As urbanization continues to surge worldwide, managing pests like mosquitoes, flies, cockroaches, and rodents has become increasingly complex. Conventional pest control methods typically rely on chemical pesticides, sparking concerns about environmental contamination, human health hazards, and the emergence of pesticide-resistant pest populations. To address these challenges, there's a growing interest in exploring alternative, environmentally friendly approaches to pest management. One such alternative gaining traction in recent years is the utilization of monoterpenes. Monoterpenes belong to a class of naturally occurring organic compounds found in various plants, notably in essential oils ([167]; H et al., 2022; [336]). They are characterized by their volatility and distinct aroma, often serving as a defense mechanism against herbivores and pathogens in plants. In the realm of urban pest control, monoterpenes have emerged as promising contenders for their potential effectiveness in repelling or disrupting the behavior of common urban pests. They offer benefits such as biodegradability, low toxicity to non-target organisms, and minimal environmental impact.

This thorough discussion aims to explore the diverse impacts of monoterpenes in urban pest control comprehensively. It will analyze how monoterpenes interact with pest organisms, their effectiveness in repelling or managing various urban pests, and the potential challenges and limitations associated with their real-world use in pest management. Furthermore, this discussion will delve into the ecological consequences of integrating monoterpenes into urban pest control strategies, considering factors such as their impact on non-target organisms, ecosystem dynamics, and long-term sustainability. Additionally, this exploration will spotlight recent advancements and innovations in applying monoterpenes for urban pest control, including formulation techniques, delivery systems, and synergistic interactions with other pest control methods. By combining findings from scientific research, field trials, and practical applications, this review aims to offer a comprehensive understanding of the role of monoterpenes in shaping the future of sustainable urban pest management practices.

## 10 Conclusion

In summary, monoterpenes serve as an exceptional illustration of nature's rich chemical arsenal, boasting a wide array of biological functions pivotal in plant defense, pest control, and ecosystem preservation. Their insecticidal, repellent, antifeedant and antimicrobial properties highlight their potential as valuable assets in integrated pest management tactics and environmentally conscious agricultural methods. As research advances in exploring the complex mechanisms behind the bioactivity of monoterpenes, their potential to tackle modern issues in agriculture, public health, and environmental conservation becomes increasingly evident. Monoterpenes, which are widely present in plants and synthesized through the terpenoid pathway—especially in structures like glandular trichomes, resin ducts, and secretory cavities—play a crucial role in plant defense. Monoterpenes act as both natural and induced defenses, protecting plants by repelling herbivores, disrupting insect feeding, and inhibiting microbial pathogens through volatile emissions. They have strong insecticidal properties, affecting key physiological functions in pests, including neurotransmission and respiration, leading to death or deterrence. Due to their selective toxicity, they target specific insect species while sparing non-target organisms, making them a promising eco-friendly alternative to synthetic insecticides. Monoterpenes repel insects through their strong odors and taste, reducing crop damage and the spread of vector-borne diseases in agriculture and forestry. They also have antimicrobial properties, fighting bacteria, fungi, and viruses, which strengthen plant resilience and help to preserve agricultural products after harvest, extending their shelf life. Monoterpenes are essential in the pathogenesis of entomopathogenic fungi (EPF), which are key biological control agents in pest management. Their interaction with EPF is complex, affecting fungal infection, growth, and enzyme production. In the environment, monoterpenes released by plants contribute to secondary organic aerosols (SOAs) through atmospheric oxidation, impacting cloud formation, climate, air quality, and tropospheric ozone. Although monoterpenes have low toxicity to humans and are useful in pest control, their broader ecological impact raises safety concerns for non-target organisms. Moreover, they offer therapeutic benefits like anti-inflammatory and antioxidant effects, but their widespread use, especially in industries like flavor and fragrance, can cause respiratory irritation and skin sensitivity. Exploring these compounds for sustainable pest management holds great promise in agriculture. By leveraging their natural properties, researchers can develop eco-friendly pest control methods that protect human health and maintain agricultural sustainability. Continued research and collaboration are essential to unlocking their full potential in IPM and supporting a more resilient food system.

## CRediT authorship contribution statement

**Muhammad Qasim:** Writing – original draft, Investigation, Conceptualization. **Waqar Islam:** Writing – original draft, Investigation. **Muhammad Rizwan:** Writing – original draft, Investigation. **Dilbar Hussain:** Writing – original draft, Investigation, Conceptualization. **Ali Noman:** Writing – original draft, Investigation. **Khalid Ali Khan:** Writing – review & editing. **Hamed A. Ghramh:** Writing – review & editing. **Xiaoqiang Han:** Writing – review & editing, Writing – original draft, Project administration, Funding acquisition, Conceptualization.

## Data available statement

No data was used for the research described in the article.

## Declaration of competing interest

All authors have no conflict of interest.
